# Application of Selective Induction Heating for Improvement of Mechanical Properties of Elastic Hinges

**DOI:** 10.3390/ma14102543

**Published:** 2021-05-13

**Authors:** Paweł Muszyński, Przemysław Poszwa, Andrzej Gessner, Krzysztof Mrozek

**Affiliations:** 1 Institute of Mechanical Engineering, Poznan University of Technology, Piotrowo 3, 61-138 Poznan, Poland; pawel.muszynski@put.poznan.pl (P.M.); andrzej.gessner@put.poznan.pl (A.G.); krzysztof.mrozek@put.poznan.pl (K.M.); 2 Institute of Materials Technology, Poznan University of Technology, Piotrowo 3, 61-138 Poznan, Poland

**Keywords:** injection molding, selective induction heating, shear rate, elastic hinge

## Abstract

Injection molding is a polymer processing technology used for manufacturing parts with elastic hinges. Elastic hinges are widely used in FMCG (Fast Moving Consumer Goods) packaging (e.g., bottle closures of shampoos, sauces) and in the electrical engineering industry. Elastic hinge is a thin film that connect two regions of the injection molded part, where significant shear rates are present, which can lead to the degradation of polymers and the decrease in mechanical properties. Selective induction heating is the method that improves the flow of the polymer melt through thin regions by the local increase in mold temperature. In this study, selective induction heating was used to improve mechanical properties of elastic hinges by the reduction of material degradation due to high shear rates. To verify the change of shear rates, selective induction heating simulation and injection molding simulations were performed. The linear relation between mold temperature and maximum shear rate in the cross-section was identified and the mechanical tests showed significant differences in hinge stiffness, tensile strength and elongation at break.

## 1. Introduction

Polymer materials are currently widely used in many industries, such as medical, electronics, automotive and building [1,2]. The popularity of polymers is associated with relatively low prices, a very good price-quality ratio and general availability [3]. Requirements for their operating, strength and aesthetic properties are growing very dynamically [4,5], motivating more and more scientists and companies to search for new manufacturing technologies or their improvements [6,7,8]. Nowadays, despite many years of usage, still the most developing technology is injection molding [9]. In this technology, the main goal is to reduce the thickness of the wall of the injection-molded part, because the thinner the wall, the shorter the cooling time can be achieved, and thus the shortening of the entire injection cycle. Reduced wall thickness results in increased injection pressure, due to the reduction in the cross-section of the mold’s cavity of the flow path of the plastic [10]. A thin layer of polymer melt cools down rapidly, which increases the resistance of the flow and results in the freezing of the material before the complete filling of the cavity [11]. The increase in the surface temperature above the transition temperature of polymer melt allows the manufacturing of long thin-walled parts. For this purpose, the Rapid Temperature Cycling (RTC) technology was developed, where the surface of the mold is periodically heated [12,13]. Higher surface temperature can significantly increase the time of part cooling, especially when technologies such as steam heating or cartridge heating are used. This is due to the fact that not only the surface but the whole mold is heated to increase surface temperature. To solve this disadvantage of increased cooling time, new techniques were developed, such as conformal cooling, algorithms that provide a better distribution of cooling channels or optimized heating procedures [14,15]. 

The application of induction heating for RTC technology has been strongly investigated in recent years because of high heating rates and small heating volume, which has led to an improvement of the process with a small increase in the cooling time. Studies in this area were carried out by Chen et al. [16,17]. New solutions were developed for this technique for better control of heating regions and even higher heating rates [18,19,20]. Menotti et al. undertake the problem of mapping the injection mold surface microstructure with use of induction heating [21]. The authors obtained the heating rate of 10 °C/s and proved that induction heating allows the improvement of the quality of manufactured parts’ surfaces. In the publication [22], the authors showed hybrid manufacturing technology that involves forming a part from two different materials. The electromagnetic induction was used to heat the metallic material, giving, in effect, a longer flow path of the melted material. The work [23] studies the influence of induction heating of the injection mold in a selected area of the cavity on the strength properties of the electrical connector housing. The authors obtained better strength properties of the material by increasing the temperature of the selected area. Giang et al. proposed the injection molding technology with assisting gas in their work [24]. The simulations and experiments were carried out in air temperatures of 400 °C and a heating time of 20 s to test a device used to focus the flow, which supports external control of the injection mold’s temperature. The authors proved that the technology can be used to improve the melt flow length in injection molding, which increased from 38.6 to 170 mm, while the balance of the melt filling was also clearly improved. The influence of infrared heating on the improvement of quality of the parts with long-fiber polymers was also studied [25]. The studies showed that the infrared heating method improved the quality of flowing polymer melts’ weld-line. Kościuszko et al. have also studied the influence of the temperature on polymeric parts’ properties [26]. The differences in the shrinkage and mechanical properties of the samples, arising from the form temperature, defined by the tensile test, were eliminated by annealing. However, the samples made with the use of two different injection mold temperatures still significantly differed in impact strength; the values were clearly higher for annealed samples in relation to the results obtained for samples right after the injection process. 

Of all the parameters, the key parameter is the temperature of the molding surfaces during the filling of the cavity by a flowing plastic melt, as many studies show [27,28,29]. 

During conventional processes of injection molding, constant-temperature injection molds are used. The difference between temperatures of flowing stream of plastic and molding surface causes the plastic melt to cool down and its viscosity to increase along with the distance traveled [30]. The formation of frozen layers reduces the cross-section of the cavity, making it impossible to fill the forming areas, which are the farthest from the injection point. Problems related to incomplete filling of the molding cavity occur especially during the processing of materials with increased viscosity or supplemented by fillers of various type (reinforcing fibers, magnetic powders, talc, flame retardants, etc.) [31]. Very often, mistakes of mapping microstructures and defects related to the inappropriate formation of weld-line of flowing melt streams accompany this phenomenon [32]. Weaknesses, which are the effect of using too low mold temperatures and increased injection pressures, can be removed in additional technological processes. However, it should be noted that from an economic and ecological point of view, it is more beneficial to carry out the complex production included in one operation of injection molding.

In the case of dynamic changes in mold temperature technology, the molding cavity does not have one constant work temperature. The temperature in the mold is changed intentionally and synchronized with the work of the injection machine, according to the profile established by the technologist. In the moment of injection, the molding surfaces are heated to the temperature close to the value of the injected plastic melt. After the injection, the intensive cooling process begins. Thus, it is possible to manufacture parts with high gloss, free from deformations and visible flowing lines of the melt and thin-walled parts, for which incomplete filling of the cavity often occurs [33]. The rheology of plastics, due to their non-Newtonian character, is directly related to the processing temperature [34]. In contrast to Newtonian liquids, the viscosity of flowing melt is not a constant value in isobaric conditions, but changes with the change of the shear rate. Techniques of cyclic regulation of the mold cavity’s temperature give manufacturers a possibility of voluntary influence on the course and the distribution of the temperature in the cavity. In turn, during the holding phase, better propagation of the pressure in the whole volume of the part occurs. Consequently, lower gradients of the pressure between the injection point and the farthest areas on the flow path of the melt appear. According to the analysis carried out by Liparoti et al., it translates into a decrease in frozen strains in the part and lower values as well as differences in the shrinkage orientation [35]. The literature is, however, in need of analyses related to selective induction heating of the molds. The relationships describing the influence of local heating of the molding cavity on the phenomena occur inside the flowing material are still not known. The authors undertake the analysis of those phenomena by realization of simulation and experimental studies. The samples were obtained by conventional injection molding and with support of the selective induction heating process. The parts were subjected to strength tests. The authors proposed the induction heating of the selected forming areas to provide a high dynamic of the heating process and short mold cooling time. During analyses, the focus was on the influence of the selective induction heating of necking areas on the shear rate of polymeric material, which translates into the strength properties of the final product.

## 2. Investigated Geometry

The use of products made from polymeric materials, which are fitted in flexible hinges, gives a lot of benefits. The hinge is a thin (0.15–0.5 mm) film between at least two parts of one plastic piece. The polymer melt flows through such a thin area and causes the orientation of polymer chains along the flowing path. That orientation is retained in the part as a result of rapid cooling. For semi-crystalline plastics, the increase in chain orientation causes the area with high crystalline degree and very high strength. The proper functioning of the hinge depends on accurate geometry. The hinge cannot be too thick, long or wide—the plastic should flow through the area of the hinge perpendicularly to its functioning axis. Since the hinge reduces the cross section through which the plastic flows, and the flow rate is determined by the thermal resistance of the plastic, this reduces the mold cavity volume behind the hinge. During the joining of the hinge parts with higher volume, the solution is to use additional injection points. In this case, the weld-line of plastic streams from different injection points cannot occur in the area of hinge forming. The durability of hinges depends on used material, the plastic part shape, construction of the mold and parameters of the injection process determined by the technologist. For semi-crystalline plastics, it can be very high (for PP it reaches 1mln of cycles), and for amorphous plastics very low—hinges are single-use, used only for installation (closing of both halves of the part).

**Figure 1:**
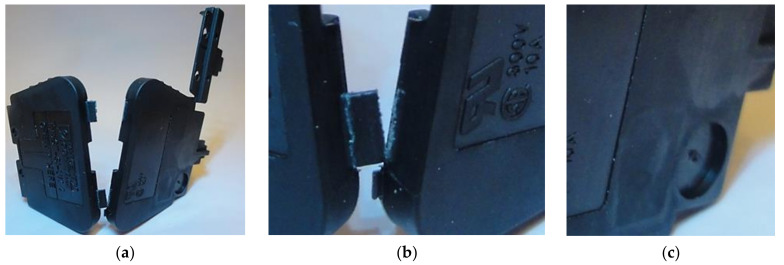
(**a**) Housing of electrical connector made from PA 66 (Celanese Frianyl A63 RV0, Irving, USA), (**b**) cracking hinges during the part closing process, (**c**) injection point.

In Figure 1, a part constituting the housing of the electrical connector is shown. The element is fitted in flexible hinges. The observed low durability of hinges in the molded parts constitutes a quality problem. 

**Figure 2:**
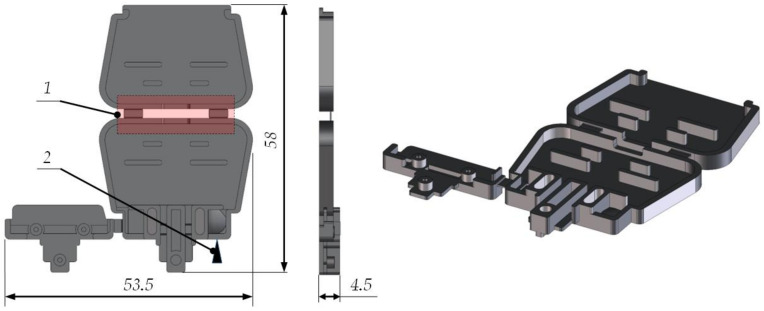
Research model of electrical connector housing, 1–forming insert of flexible hinges, 2–injection point.

In order to better understand the processes occurring during the material flow in the forming cavity, a molded part was designed for testing (Figure 2). The part was free of micro-features disrupting the creation of the mesh for the purpose of the simulation studies. Moreover, it was designed to reduce the production costs of the injection mold, which allows the manufacture of parts conventionally and with the use of the selective induction heating processes. 

Use of the parts made from polymeric materials, which are fitted in flexible hinges, gives a lot of benefits. The hinge is a thin (0.15–0.5 mm) film between at least two parts of one plastic part. The flow of the plastic melt through such a thin area causes a dynamic increase in the shear rate, which in turn results in the degradation and weakening of the strength properties of the final product.

## 3. Experimental Setup

### 3.1. Selective Induction Heating Simulations

In the present article, simulation studies of selective induction heating of injection molds were conducted. The simulation analysis was carried out by using the Finite Element Method (FEM) implemented in the QuickField 6.3.1 package (Tera Analysis, Svendborg, Denmark). All of the tests were performed in 2D (XY) space in AC Magnetics modules (electromagnetic analysis) to determine the current density on the surface of the metal insert, followed by transient heat transfer (thermal analysis) to determine cavity surface temperature $T_{mold}$ as a function of time.

The analyses was performed for steel 1.2343 and its properties are presented presented in Table 1.

### 3.2. Thermal Measurements

To validate the results obtained with numerical simulations of selective induction heating, the thermal camera FLIR T620(FLIR, Wilsonville, OR, USA) was used. 

### 3.3. Injection Molding Simulations 

The injection molding simulations were performed with Autodesk Moldflow Insight 2019 (Autodesk, San Rafael, CA, USA) software, which uses a Cross-WLF thermorheological model to describe the behavior of polymer melt during the filling of the cavity (it is the material model that is widely used for injection molding simulations [36]). The mesh size in the whole part was set to 0.5 mm, whereas the dense mesh was created at the elastic hinges (mesh size of 0.1 mm and the number of layers was increased from 10 to 20) to obtain more accurate results.

In this study, the influence of different injection times ($t_{inj}\;\in \left\{0.45,\;0.55,\;0.65,\;0.75,\;0.85\right\}\;\left[s\right]$) and different surface temperatures ($T_{mold}\in \left\{80,\;130,\;150,\;170,\;190\right\}\;\left[℃\right]\;$) on the maximum shear rate were investigated. The $T_{mold}$ higher than 80 ℃ was obtained using selective induction heating. The achievable temperature was determined according to results from selective induction heating simulations. In this study, the defined $T_{mold}$ was set in hinge region for whole $t_{inj}$.

The results from the simulations were extracted with the script prepared in VBScript language to minimize the probability of measurement error. The script adds XY Probe plots that read specific values through the thickness of the part at specific locations. In this study, nine measurement points evenly distributed at both hinges were used. After the plot is added, the software exports the values in specific points through the thickness of the part in the *.txt file.

The obtained results were merged in *.xlsx spreadsheet and then the data were visualized with a script prepared in Python language (pandas and matplotlib libraries were used) [37,38,39,40,41]. This was done because of the complicated structure of the obtained data (every result measured through the thickness of the part was composed of several dozen points).

### 3.4. Mechanical Tests

In this study, the mechanical strength of elastic hinges was investigated by a tensile test. The experiment was performed with Instron 4481(ISO 527), where the constant rate of elongation was set to 1 mm/s. The specimens manufactured with $T_{mold}=130\;℃$ and $t_{inj}=0.65\;s$ were cut in half and both hinges were investigated separately.

## 4. Results and Discussion

**Figure 3:**
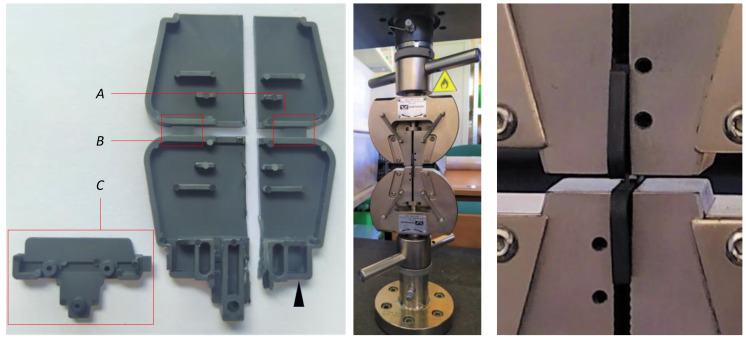
A test sample cut in half and mounted on a testing machine. (**A**) The hinge located closer to the injection point, (**B**) the second hinge, (**C**) separated part of the molding.

**Figure 4:**
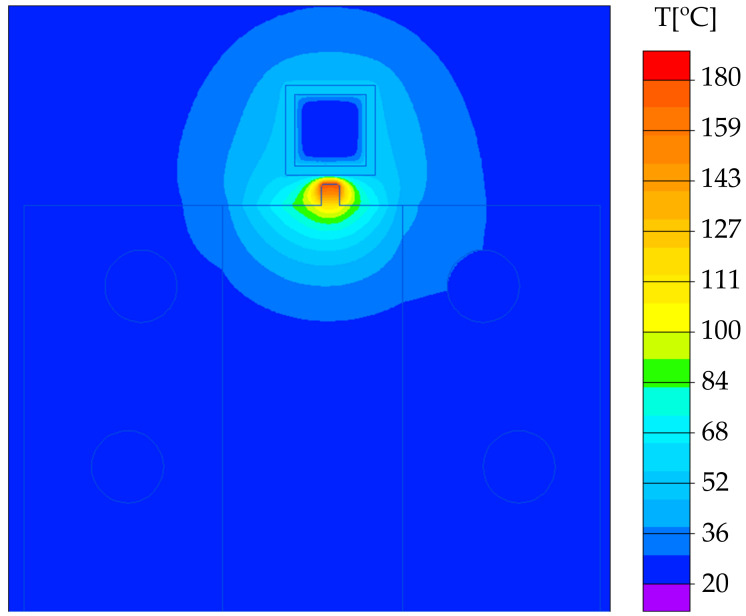
The temperature of the selectively heated region after 2.5 s.

**Figure 5:**
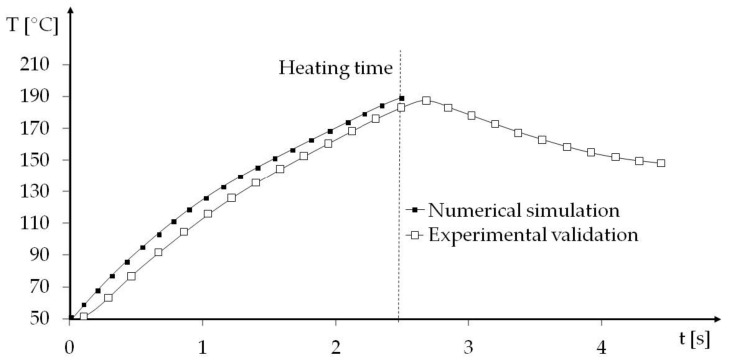
The temperature in the middle of the hinge (numerical and experimental results).

The numerical simulation of induction heating is presented in Figure 3 and Figure 4. The first diagram presents the temperature distribution in the selectively heated hinge region after 2.5 s—the temperature in the hinges’ region is even. The temperature in the center of the hinge was measured in time (Figure 4), and after 2.5 s it reached ~190 ℃—that value was set as maximum $T_{mold}$. In Figure 5, the experimental validation of the simulated phenomenon is shown.

**Figure 6:**
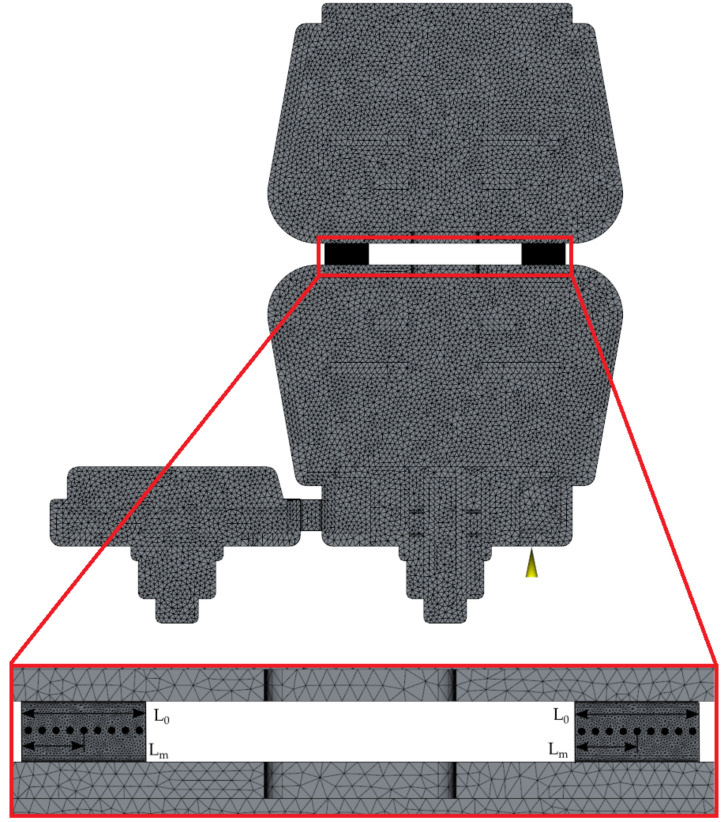
The measurement points for the shear rate evaluation placed on a hinge farther from (**right**) and closer to (**left**) the injection point.

The measurement was taken in nine points in the middle of the hinge (Figure 6) and the values of the shear rate presented in the diagrams are the maximum values of shear rate through the whole filling phase

**Figure 7:**
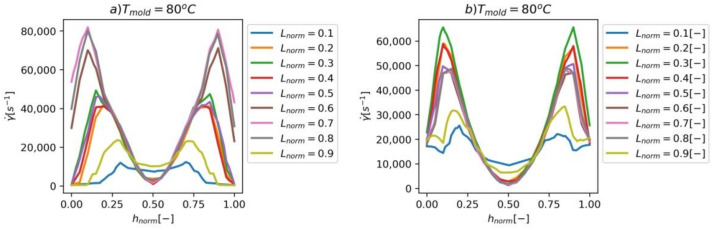

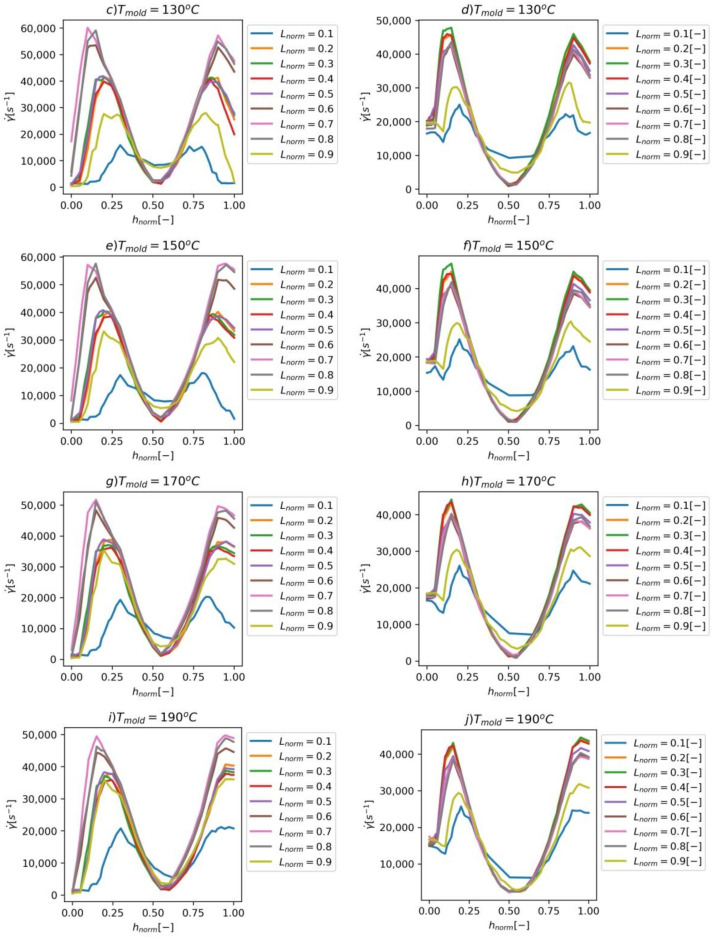
(**a**–**j**) The shear rate $\dot{\gamma }$ through the thickness h of both hinges (**left**—the hinge closer to the injection point, **right**—the hinge farther from the injection point). The results were obtained for $t_{inj}=0.65\;s$.

In Figure 7a–j are results of shear rate $\dot{\gamma }$ through the normalized thickness $h_{norm}$ of both hinges. The specific curves present shear rate values at different locations defined by normalized width $L_{norm}=\frac{L_{m}}{L_{0}}$, where $L_{m}$ is the distance from the edge of the hinge and $L_{0}$ is the width of the hinge. The diagrams present the shear rates $\dot{\gamma }$ for different cavity surface temperatures, $T_{mold}$. When $T_{mold}=80\;℃$, the diagrams are symmetrical as the $T_{mold}$ for both sides of the hinges are equal. When the temperature is increased via selective induction heating, the asymmetry in shear rate distribution is present. There is a significant difference (10,000–15,000 $s^{-1}$) in values of shear rate for both hinges—the hinge that is closer to the injection point has a higher shear rate, because of the higher flow rate of the polymer melt in that hinge.

**Figure 8:**
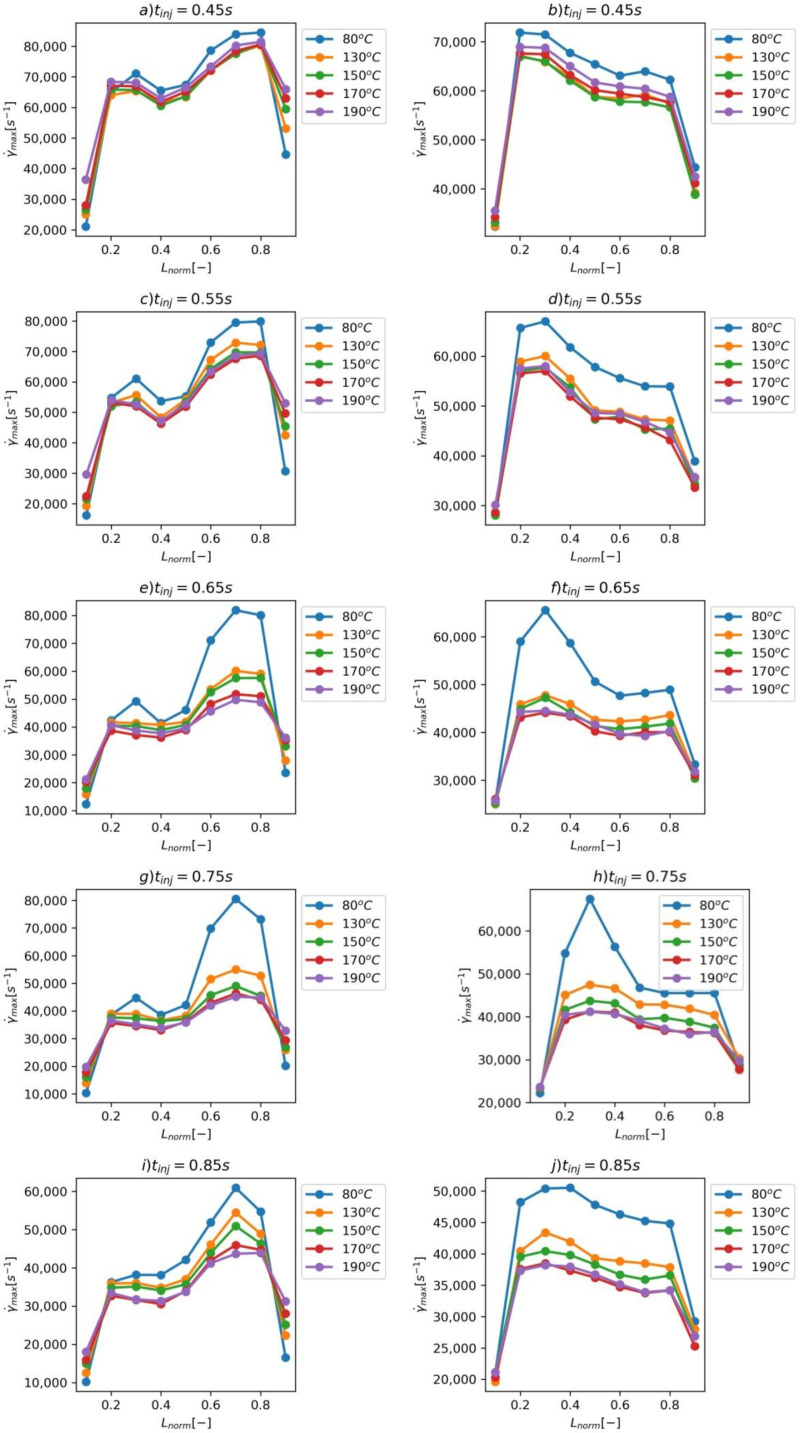
(**a**–**j**) The maximum shear rate ${\dot{\gamma }}_{max}$ through the normalized width of the hinge $L_{norm}$ of both hinges (**left**—the hinge closer to the injection point, **right**—the hinge farther from injection point).

In Figure 8a–j are the results of the maximum shear rate ${\dot{\gamma }}_{max}$ through the normalized length $L_{norm}$ for both hinges. The diagrams present results for different $T_{mold}$ and $t_{inj}$. The maximum obtained is nonevent through $L_{norm}$. At the edges of the hinge, the shear rate is smaller because of the closeness to the wall. The differences in ${\dot{\gamma }}_{max}$ in the hinge results from the different local velocity of polymer melt and are equal to 10,000–30,000 $s^{-1}$. The most significant differences are present for $T_{mold}=80\;℃$—the increase in the $T_{mold}$ provides more uniform ${\dot{\gamma }}_{max}$ distribution through the $L_{norm}$. There is also a difference in ${\dot{\gamma }}_{max}$ between hinges equal to 10,000–15,000 $s^{-1}$.

The next step was to investigate the relation between ${\dot{\gamma }}_{max}$, $t_{inj}$ and $T_{mold}$. For $t_{inj}$, it was impossible to find a particular relation, as local velocity changes with $L_{norm}$ and $t_{inj}$. Fortunately, a linear relationship between ${\dot{\gamma }}_{max}$ and $T_{mold}$ is present. The verification was done with SciPy library for Python programming language that contains linear regression module and Equation (1) was obtained [40]:

(1) $${\dot{\gamma }}_{max}=a_{{\dot{\gamma }}_{max}\;}T_{mold}+b_{{\dot{\gamma }}_{max}}$$

**Figure 9:**
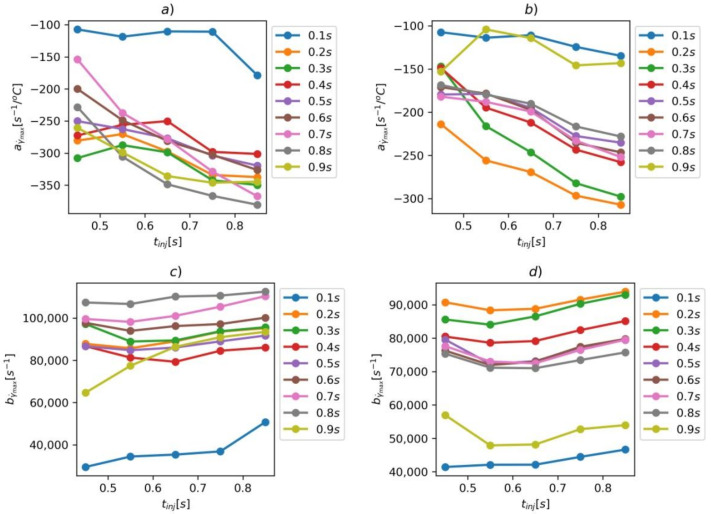
(**a**–**d**) The slope $a_{{\dot{\gamma }}_{max}\;}$ and intercept $b_{{\dot{\gamma }}_{max}}$ in function of $t_{inj}$ for different $L_{norm}$ (**left**—the hinge closer to the injection point, **right**—the hinge farther from the injection point).

According to obtained results for 90 measurement points (45 per hinge), 86 of fitted lines had a $R^{2}<-0.95$ (−0.978±0.012). The remaining four measurements were removed for further analysis as the numerical error. The shape of the slope $a_{{\dot{\gamma }}_{max}\;}$ and intercept $b_{{\dot{\gamma }}_{max}}$ are presented in Figure 9a–d.

The differences in the range of $a_{{\dot{\gamma }}_{max}\;}$,$\;b_{{\dot{\gamma }}_{max}}$ values are not very significant (the bigger difference is present for $b_{{\dot{\gamma }}_{max}}$). The values of both coefficients are directly related to the local velocity of polymer melt and the values are in the range: $a_{{\dot{\gamma }}_{max}\;}\in \left(-375;-100\right)\left[\frac{s^{-1}}{℃}\right]$ and $b_{{\dot{\gamma }}_{max}\;}\in \left(30,000;11,500\right)\left[s^{-1}\right]$. The $a_{{\dot{\gamma }}_{max}\;}$ value drops with the increase in $t_{inj}$ and $b_{{\dot{\gamma }}_{max}\;}$ slightly rises with the increase in $t_{inj}$. The location of the measurement point had the most significant influence in this case. If the extreme measurement points were removed, the values would be in the range: $a_{{\dot{\gamma }}_{max}\;}\in \left(-375;-150\right)\left[\frac{s^{-1}}{℃}\right]$ and $b_{{\dot{\gamma }}_{max}\;}\in \left(70,000;11,500\right)\left[s^{-1}\right]$, so only the extreme points have a significant influence on $b_{{\dot{\gamma }}_{max}\;}$.

The last step was the verification of the mechanical properties of the elastic hinges. The measurements were performed for each hinge separately (the plastic part was cut in half and tested). The results are presented in Table 2.

The obtained stiffness modulus was higher than the data presented in the technical datasheet, while the elongation at break was significantly lower. The difference in those parameters results from the orientation of polymer chains during the flow through the thin region (due to high shear stress). Unfortunately, for the hinge closer to the injection point, the shear stress was too high and the beneficial effect of material orientation was limited, especially for stiffness modulus E and tensile strength $R_{m}$.

## 5. Conclusions

In this work, the possibility of applying selective induction heating to improve the mechanical properties of elastic hinges was investigated. In the first step, the achievable cavity surface temperature was determined, where ~190 °C was observed in 2.5 s. The next step was to investigate the filling of the cavity by polymer melt to determine the magnitude of shear rate that can lead to the polymer degradation.

The polymer grade (Celanese Frianyl A63 RV0, Irving, TX, USA) used in this study has a maximum shear rate ${\dot{\gamma }}_{max}$ equal to 60,000 $s^{-1}$. For $t_{inj}>0.45\;s$, the application of selective induction heating was sufficient to reduce the maximum shear rate below the critical value.

To verify the benefits of the selective induction heating, the tensile test was performed for the parts manufactured where ${\dot{\gamma }}_{max}$ was below the critical value. Both hinges were manufactured with different ${\dot{\gamma }}_{max}$ due to differences in local velocity of the polymer melt. It resulted in the difference in ${\dot{\gamma }}_{max}$ equal to 10,000 $s^{-1}$ which lead to stiffness modulus *E*, tensile strength $R_{m}$ and elongation at break ε being lowered by 15%, 31% and 24%, respectively.

The results indicate that further increases of cavity surface temperature can significantly improve the mechanical performance of elastic hinges due to further reduction of the maximum shear rate to values that left beneficial effects of polymer chain orientation.

## Figures and Tables

**Table 1:** Properties of the steel used in the analysis.

Property	Steel 1.2343
Relative magnetic permeability *μ*_r_	55
$\mathrm{Electrical}\;\mathrm{conductivity}\;\sigma \;\left[\frac{S}{m}\right]$	1e8
Thermal conductivity K [$\frac{W}{mK}$]	45
$\mathrm{Density}\;\rho \;[\frac{g}{cm^{3}}$]	7.8
$\mathrm{Specific}\;\mathrm{heat}\;C\;[\frac{J}{kgK}$]	460

**Table 2:** Mechanical properties of investigated hinges.

Property	Farther Hinge	Closer Hinge	Technical Datasheet
Stiffness modulus E [MPa]	4491±171	3837±55	3600
$\mathrm{Tensile}\;\mathrm{strength}\;R_{m\;}\left[\mathrm{MPa}\right]$	91.4 ± 6.1	62.8±2.3	85
Elongation at break ε [%]	2.28±0.1	1.73±0.08	10

## Data Availability

Data is contained within the article or supplementary material.

## Supplementary Materials

The following are available online at https://www.mdpi.com/article/10.3390/ma14102543/s1, Excel Files.
